# Temporal Trends and Spatial Variability of Vegetation Phenology over the Northern Hemisphere during 1982-2012

**DOI:** 10.1371/journal.pone.0157134

**Published:** 2016-06-08

**Authors:** Siyuan Wang, Bojuan Yang, Qichun Yang, Linlin Lu, Xiaoyue Wang, Yaoyao Peng

**Affiliations:** 1 Key Laboratory of Digital Earth, Institute of Remote Sensing and Digital Earth, Chinese Academy of Science, Beijing, China; 2 Ecosystem Dynamics and Global Ecology Laboratory, School of Forestry and Wildlife Sciences, Auburn University, Auburn, Alabama, United States of America; Kerala Forest Research Institute, INDIA

## Abstract

Satellite-derived vegetation phenology has been recognized as a key indicator for detecting changes in the terrestrial biosphere in response to global climate change. However, multi-decadal changes and spatial variation of vegetation phenology over the Northern Hemisphere and their relationship to climate change have not yet been fully investigated. In this article, we investigated the spatial variability and temporal trends of vegetation phenology over the Northern Hemisphere by calibrating and analyzing time series of the satellite-derived normalized difference vegetation index (NDVI) during 1982–2012, and then further examine how vegetation phenology responds to climate change within different ecological zones. We found that during the period from 1982 to 2012 most of the high latitude areas experienced an increase in growing period largely due to an earlier beginning of vegetation growing season (BGS), but there was no significant trend in the vegetation growing peaks. The spatial pattern of phenology within different eco-zones also experienced a large variation over the past three decades. Comparing the periods of 1982–1992, 1992–2002 with 2002–2012, the spatial pattern of change rate of phenology shift (RPS) shows a more significant trend in advancing of BGS, delaying of EGS (end of growing season) and prolonging of LGS (length of growing season) during 2002–2012, overall shows a trend of accelerating change. Temperature is a major determinant of phenological shifts, and the response of vegetation phenology to temperature varied across different eco-zones.

## Introduction

Vegetation phenology is highly sensitive to climate change, so monitoring vegetation phenology is becoming an increasing important way to understand and quantify global environmental changes [1, 2]. Since the 1950s, the world’s mean surface temperature has increased by 0.6°C, and the warming trend has been faster in the high latitude areas, such as Eurasian continent [3]. As a result of the recent increase in surface temperature, earlier spring phenological events have been investigated in the northern hemisphere [1], [2], [4]. Shifts in vegetation phenology will affect ecosystem functions since they affect hydrological cycle, surface energy balance, and terrestrial carbon cycle [5–6]. Therefore, it is important to improve our understanding to quantify accurately the timing of phenological shifts for improving our understanding of terrestrial ecosystems response to global change.

In the past few decades, satellite remote sensing has been recognized as a valuable tool for acquiring and analyzing information about land surface development. In particular, time series of remote sensing data are an important data source for investigating vegetation phenological changes. The seasonal dynamics in a long-term vegetation index (VI) time series such as those from the AVHRR, SPOT, or MODIS sensors can be used for plant phenological detection. For example, time series of NDVI data derived from AVHRR or MODIS data have been used to estimate the inter-annual variations in vegetation phenology for the past decades [7–8]. Annual time series of Fraction of Absorbed Photosynthetically Active Radiation (FAPAR) data have been used to identify key phenological events [9]. Recent, research efforts have been focused towards improving algorithms for calculating vegetation phenological shifts using other satellite-derived data (e.g. MODIS-enhanced vegetation index (EVI), leaf area index (LAI), FAPAR and Albedo) and modeling techniques.

Many previous studies investigated the timing of phenological events and their connection to climate change by using time-series of remote sensing data in the northern hemisphere [2],[10–15]. Due to the increase in land surface temperature, strong vegetation phenology shifts have occurred in the northern hemisphere. For example, in North America, a delayed dormancy onset date (0.551 days yr^-1^) and an extended growing season length (0.683 days yr^-1^) were observed over the mid and high latitudes during 1982–2006 [16]. In China, the beginning of the growing season (BGS) has advanced in spring by 0.79 days yr^-1^ and the end of the growing season (EGS) delayed in autumn by 0.37 days yr^-1^ in temperate regions during 1982–1999 [17]. In Europe, an earlier BGS (0.54 days yr^-1^) and a prolonged length of vegetation growing season (LGS) (0.96 days yr^-1^) were found during 1982–2001 [18]. For the Northern Hemisphere as a whole, BGS advanced by 5.2 days during 1982–1999 but advance by 0.2 days during 2000–2008, and EGS was delayed by 4.3 days in the period of 1982–1999 and it was further delayed by another 2.3 days in the period of 2000–2008 [2]. However, although it took a great deal of effort to examine phenological shifts over the northern hemisphere, great uncertainties still exist. Just as our literature review revealed that many studies present different magnitudes of phenological shifts, including earlier or later phenology events and longer or shorter vegetation growing seasons [13], [19–21]. Furthermore, some other studies only concentrated on earlier BGS or assessed later shifts of dormancy events [9], [22–24].

In this paper, we intend to investigate the spatial variability and temporal trends of vegetation phenology shifts in the northern hemisphere during 1982–2012, and further examine the mechanisms of phenology response to climate change within different ecological zones. Our objectives in this study are to better understand the following key scientific questions: (1) What is the vegetation phenology pattern on the pixel level in the northern hemisphere? (2) How did the patterns of phenology shift within different ecological zones over the past three decades? And (3) what are the key factors affecting vegetation growing season change in recent years? Answers to these questions are essential to improve our understanding of phenological shifts caused by climate warming and to formulate measures for climate warming and environmental problems at the global level.

## Materials and Methods

### Data Source and Pre-Processing

#### NDVI dataset from GIMMS

The NDVI_3g_ data with a spatial resolution of 8 km×8 km and a temporal resolution of a 15-day interval were obtained from the NASA Global Inventory Modeling and Mapping Studies (GIMMS) group. Data were derived from the AVHRR instrument onboard the NOAA satellite series 7, 9, 11, 14, 16, and 17 for the time period of January 1982 to December 2011, and these data had been corrected for calibration, solar geometry, heavy aerosols, clouds and other effects not related to vegetation change [25–27].

#### NDVI dataset from MODIS

The Moderate Resolution Imaging Spectroradiometer (MODIS) onboard the Terra and Aqua satellites provide near-daily repeated coverage of the earth’s surface with 36 spectral bands and a swath width of approximately 2330 km. For this study we selected the MOD 13C1 for global vegetation information extraction. The MODIS products are distributed through NASA’s Earth Observing System Data and Information System (EOSDIS). The MODIS product (MOD 13C1), with a 0.05°spaital resolution and 16-d intervals from 2002~2012, was generated from atmospherically corrected by using bi-directional surface reflectance functions, and masked for water, ices, clouds, aerosols, and shadows [28–29].

#### Holdridge life eco-zones data

**Fig 1:**
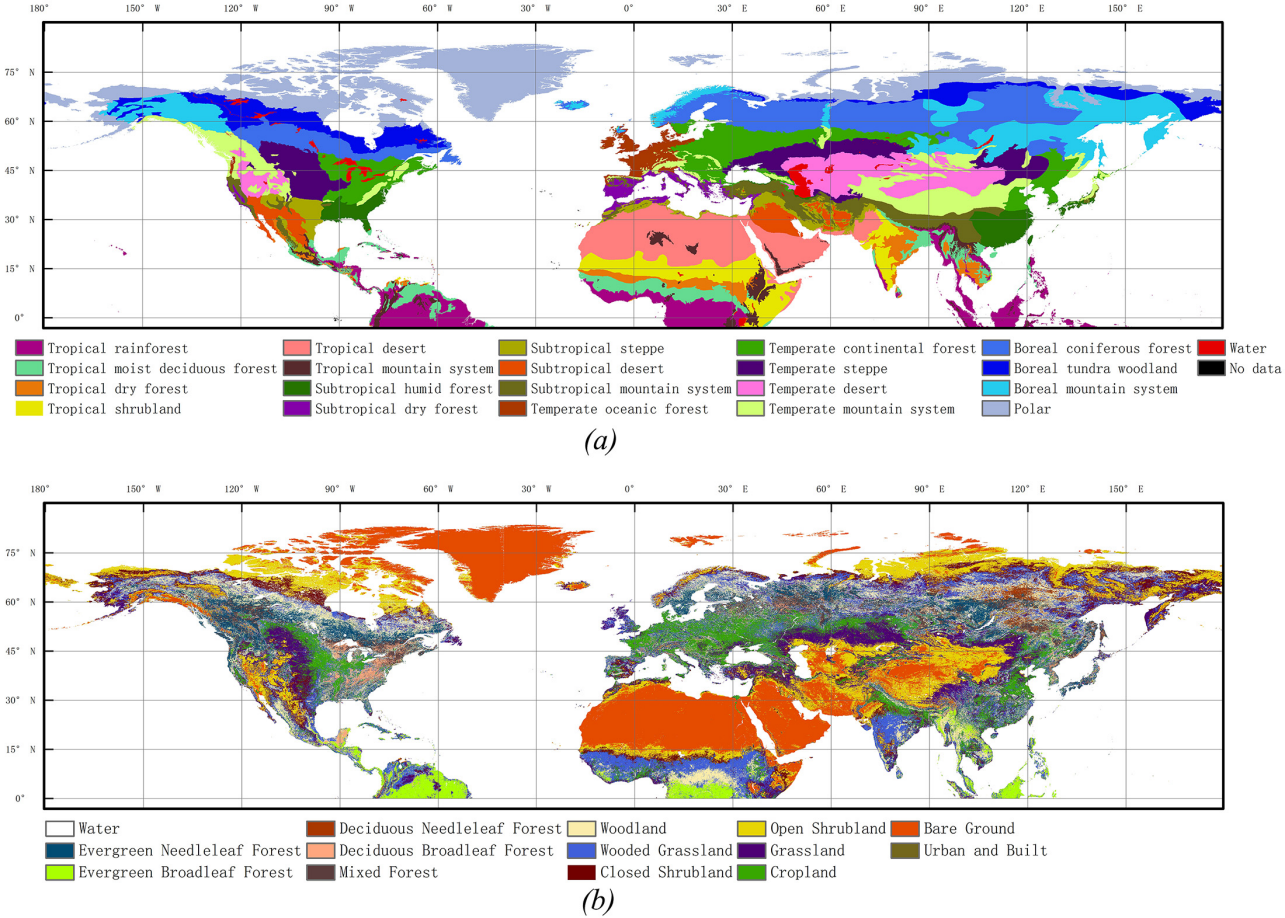
**(*a*) Spatial pattern of the Holdridge life eco-zones in the Northern Hemisphere. (*b*) Distribution of vegetation types in the Northern Hemisphere** (adapted from UMD Global Land Cover Classification, 1 Kilometer, Department of Geography, University of Maryland).

The Holdridge life eco-zone is one of the most widespread, quantitative schemes for classification of vegetation, land use, and environments.The concept of life zones was first published by Leslie Holdridge in 1947, and can be used to determine land use options and ecological types. The Holdridge life eco-zones data set was acquired from the Food and Agriculture Organization of the United Nations (FAO). The data set shows the Holdridge life eco-zones of the world, a combination of climate and vegetation types. The life eco-zones were devised using three indicators: mean annual biotemperature (based on the growing season length and temperature), mean annual precipitation (including rain and snow), and a potential evapotranspiration ratio [30–31]. The data set has a spatial resolution of one-half degree latitude/longitude, and a total of 20 life eco-zones, ranging from the evergreen tropical rainforest zone to the boreal tundra woodland zone (Fig 1).

#### Global meteorological data set

The global meteorological data set was acquired from the National Centers for Environmental Prediction/National Center for Atmospheric Sciences (NCEP/NCAR). The NCEP/NCAR reanalysis meteorological data have been widely used to study and evaluate vegetation phenology. The NCEP/NCAR Reanalysis project incorporates observations and numerical weather prediction (NWP) model output from 1948 to present. Results are available at 6 hour intervals (4-times daily), and two variables were acquired for this study: mean temperature and mean rainfall. Time series are composed of 10-day global images at 0.5 degree resolution.

#### Global vegetation cover data Set

The global vegetation cover characteristics data were acquired from Department of Geography, the University of Maryland [32–33]. The global vegetation classification data set, with 1 kilometer pixel resolution, was generated from 1-km AVHRR imagery data. Imagery from the AHHRR satellites acquired between 1981 and 2000 were analyzed to distinguish the following thirteen land cover types: evergreen needleleaf forest, evergreen broadleaf forest, deciduous needleleaf forest, deciduous broadleaf forest, mixed forest, woodland, wooded grassland, closed shurbland, open shrubland, grassland, crop land, bare ground, urban and built (Fig 1).

#### Preprocessing of NDVI time-series data

GIMMS NDVI_3g_ and MODIS NDVI datasets were processed to have uniform spatial projection, and to reduce the cloud and atmospheric effects before using them. The maximum value composition (MVC) was applied to the MODIS NDVI data and the AVHRR NDVI time series for cloud filtering. However, there was still some cloud contamination existing in some cases. To eliminate errors caused by clouds, snow and ice contamination, we applied a Savitzky-Golay filtering procedure to each annual NDVI cycle for smoothing and reconstrucing the NDVI time series, as described by *Chen et al*. [34] and *Broich et al*. [35]. For MODIS MOD13C1 product, a summary pixel reliability layer has been included in the MOD13C1, we first use the reliability layer information to locate the cloud or ice/snow pixels (where the pixel value equals 2 or 3 in the reliability layer) in the NDVI time-series. Then these cloud or ice/snow pixels can be replaced by a linear interpolation method using the adjacent NDVI data. The new NDVI values can be calculated as follows:

$$NDVI^{N}\left(i,t\right)=\;\{\begin{array}{ll} aNDVI\left(i,t-1\right)+bNDVI\left(i,t+1\right), & PR=2\;or\;PR=3\\ NDVI\left(i,t\right), & PR=0\;or\;PR=1 \end{array}$$ (1)

Where *PR* (Pixel Reliability) is the value of pixel reliability and *NDVI(i*, *t)* is the value of NDVI of the *i*th pixel in the *t*th time.

To further minimize the influence of soil and non-vegetated signals in NDVI time series, we chose targeted study regions with multiyear average NDVI greater than 0.1 in the Northern Hemisphere based on GIMMS (1982–2006) and MODIS (2002–2012) NDVI data.

### Determination of Phenological Parameters

**Fig 2:**
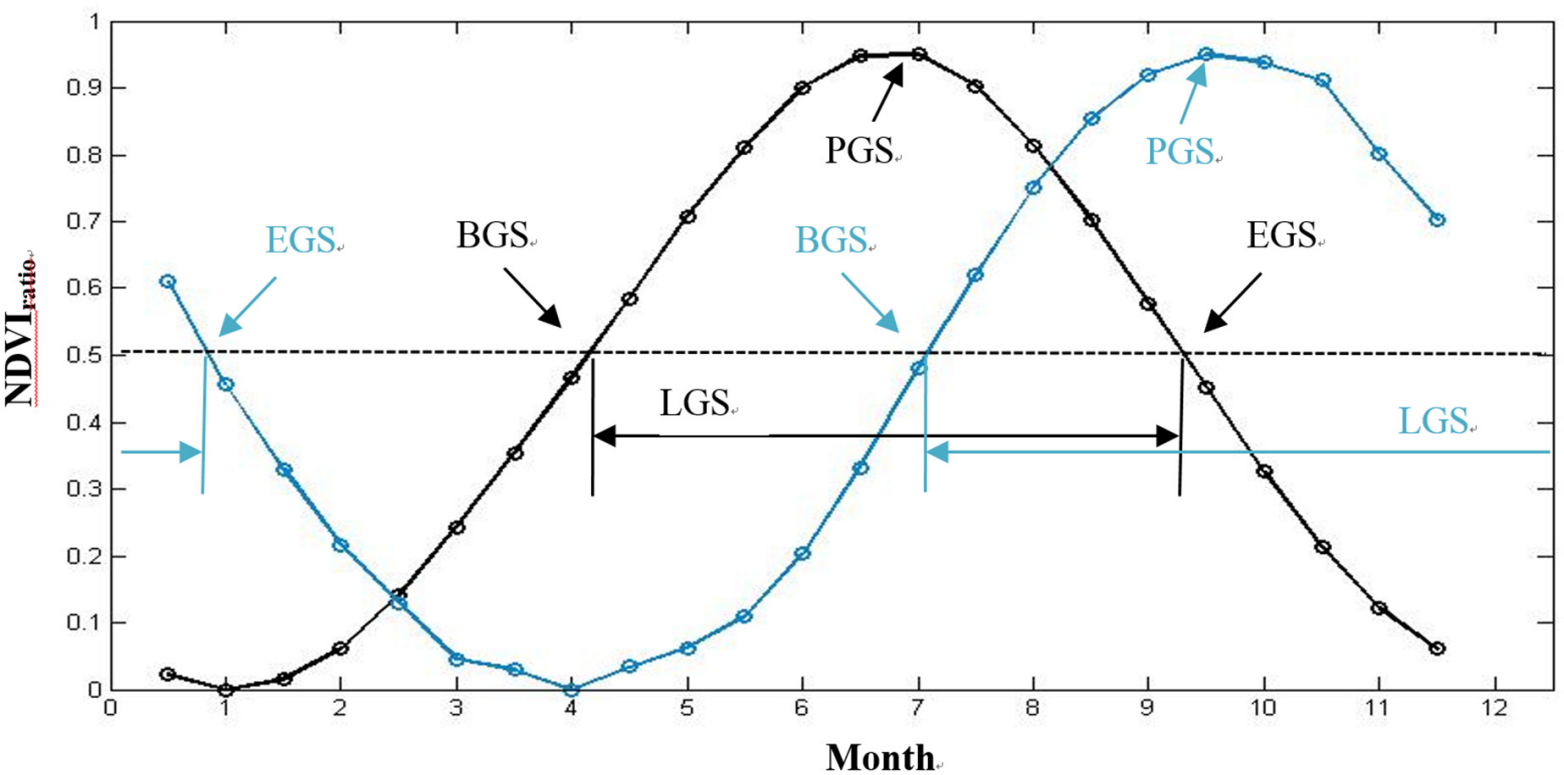
Schematic figures illustrating method identifying the beginning of growing season (BGS), end of growing season (EGS), length of growing season (LGS) and peak of growing season (PGS).

Several methods have been developed to detect the phenophases (greenup, maturity, senescence, and dormancy) of vegetation phenology by using NDVI data, such as NDVI thresholds [7, 36], largest NDVI increase [37], backward-looking moving averages [38], and fitting logistic functions [38–39]. Comparative studies have shown that the Midpoint_pixel_ threshold method based on variations of an NDVI_ratio_ is the one of the most consistent methods for the estimation of the BGS, EGS, LGS and peak of growing season (PGS) over a variety of ecosystems and corroborates with ground-based phenology data [7]. Thus, for the determination of BGS, EGS based on NDVI, we used the Midpoint_pixel_ threshold method, where the value midpoint was set at fifty percent of the maximum NDVI [7]. Firstly we calculated the annual NDVI ratio time series curve during 1982–2012. Subsequently, the phenology date was determined for each pixel by using the midpoint pixel method, and BGS or EGS was determined according to the slope of the fitted curve. Fig 2 shows the general scheme of the method for determining the phenological parameters. The NDVI ratio was calculated by using the following formula:

$$NDVI_{ratio}=\;\frac{NDVI-NDVI_{\text{min}}}{NDVI_{max}-NDVI_{min}}$$ (2)

Where *NDVI*_*r*atio_ is the output ratio, ranging from 0–1, *NDVI* is the daily NDVI, *NDVI*_max_ is the annual maximum NDVI, and *NDVI*_min_ is the annual minimum NDVI. Thus the phenological parameters can be extracted with a Midpoint_pixel_ thresholds method for each pixel.

### Phenology Trend Analysis

The spatial distribution of the phenology shift trends in the Northern Hemisphere from 1982–2012 based on the GIMMS and MODIS NDVI datasets was characterized by the change rate of phenology shift (RPS), which was calculated by the following relationship:

$$RPS=\frac{n\times \sum_{i=1}^{n}i\times Day_{i}-\sum_{i=1}^{n}i\sum_{i=1}^{n}Day_{i}}{n\times \sum_{i=1}^{n}i^{2}-{\left(\sum_{i=1}^{n}i\right)}^{2}}$$ (3)

Where *i* is the year for 1 to *n*; *n* is the total number of years; and *Day*_*i*_ is the seasonal phenology (BGS, EGS, LGS and PGS) of year *i*. A positive value of *RPS* indicates an increase of phenology in a time series. The timings of these parameters were calculated to the day because we took the day of the satellite observation into account.

Correlation coefficients and significance tests were used to examine the ralationship betweeen climate and phenology shift over the period of analysis, 1982–2012. We analysed the effects of seasonal temperature and precipitation on the trends of phenology shift in different Holdridge life ecozones by Pearson correlation analysis. In addition, a *t*-test determined the significance of the correlation, with *p* values <0.1, 0.05 and 0.01 are considered to be low, middle and high significance levels, respectively.

## Results and Analyses

### Spatial Patterns of Vegetation Phenological Metrics

**Fig 3:**
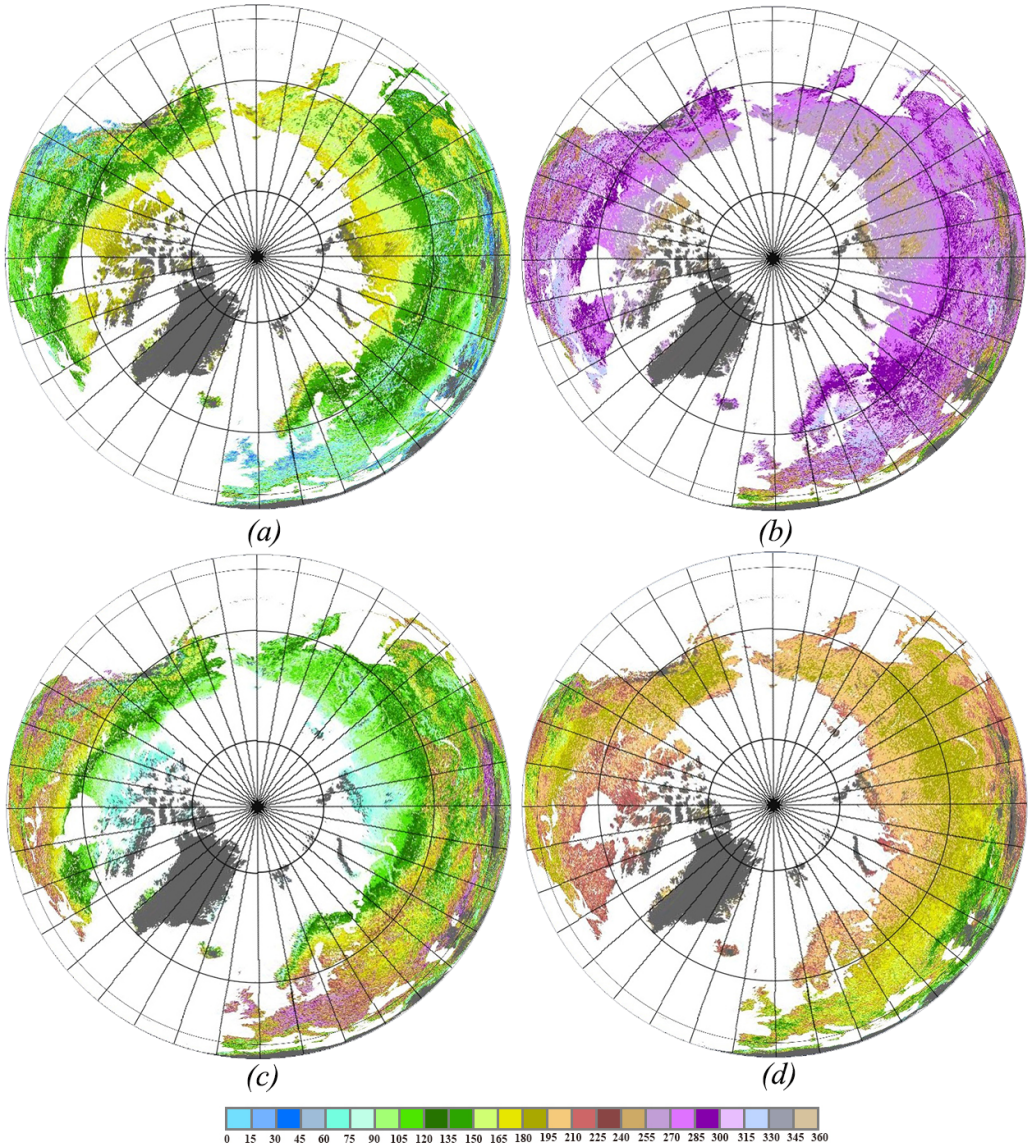
Spatial distributions of the mean (*a*) BGS, (*b*) EGS, (*c*) LGS and (*d*) PGS in the northern hemisphere during 2002–2012. The results were calculated by 11-year MODIS NDVI data.

Fig 3 shows the spatial distributions of the phenological metrics BGS, EGS, LGS and PGS over the northern hemisphere. The BGS dates ranged widely from approximately 15 days (15 January) at low latitudes to approximately 225 days (15 July) at high latitudes. The mean BGS date was progressively delayed with increasing latitude. Concurrently, the progressive patterns that vegetation turn green gradually along latitudinal gradient are mainly attributed to the climatological temperature [2], [40]. The BGS in Eurasia was much earlier than in North America at the same latitude, and the latest dates of BGS occured in northern Canada and northern Siberia owing to low temperatures. The EGS dates ranged from 15 May to approximately 30 November, the LGS value ranged from 64 days to 240 days, and the PGS value ranged from 64 days to approximately 240 days. There were opposite spatial patterns between BGS and EGS over the northern hemipshere. The dates of vegetation senescence occured in reverse order of the green-up onset. Compared with other regions in the hemisphere, east Asia, Europe and southern North America had earlier BGS and later EGS, and thus had longer growing seasons, with some regions lasting up to 10 months. In contrast, in northern Canada and northern Siberia the vegetation growing seasons lasted only 1 month.

### Spatial and Temporal Variations of Phenological Metrics over the Northern Hemisphere

**Fig 4:**
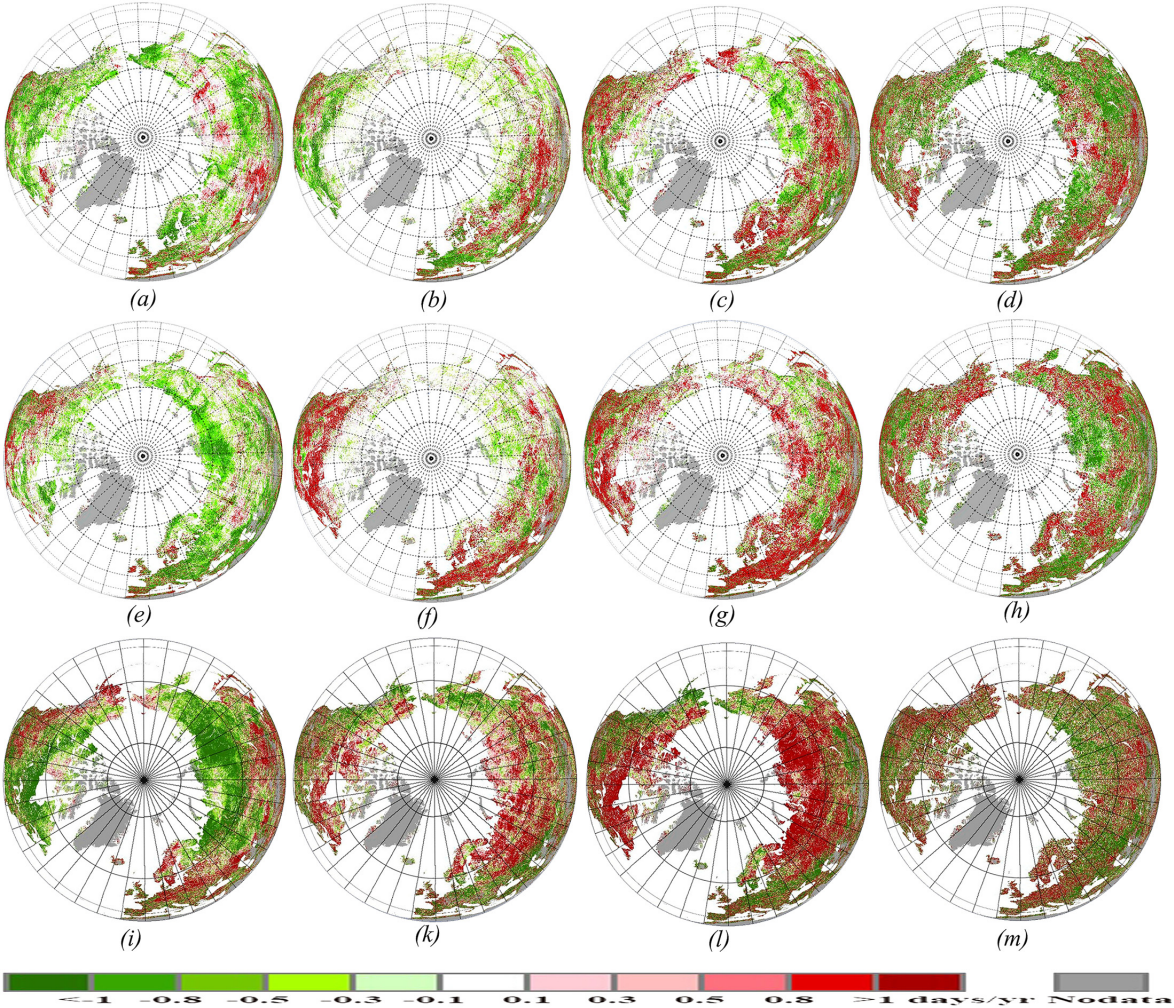
Spatial distribution of the changes in dates of BGS, EGS, LGS and PGS over the past three decades in the Northern Hemisphere. Positive values (red colors) represent later onset (BGS), later finish (EGS), longer duration (LGS) and later peak of the growing season. (*a*), (*e*), (*i*): changes in dates of vegetation green-up for 1982–1992, 1992–2002 and 2002–2012, respectively (days yr^-1^). (*b*), (*f*), (*k*): changes in dates of vegetation senescence for 1982–1992, 1992–2002 and 2002–2012, respectively (days yr^-1^). (*c*), (*g*), (*l*): changes in dates of vegetation growing season length for 1982–1992, 1992–2002 and 2002–2012, respectively (days yr^-1^). (*d*), (*h*), (*m*): changes in dates of vegetation growing peak for 1982–1992, 1992–2002 and 2002–2012, respectively (days yr^-1^).

The spatial pattern of the changes of BGS, EGS, LGS and PGS for periods of 1982–1992, 1992–2002 and 2002–2012 are mapped in Fig 4. There were clear temporal shifts of phenology metrics (BGS, EGS, LGS and PGS) from 1982 to 2012. During the entire period of 1982–2012, many high-latitude regions showed a trend towards earlier BGS and delayer EGS, resulting in a longer LGS, but trends in PGS show a more fragmented pattern than BGS, EGS and LGS. During 1982–1992, more than 80% of earlier BGS (over 0.5 days yr^-1^) mainly occurred in northern America and Eurasia, with exceptions being parts of Russia, which showed a delayed BGS (Fig 4*a*). A slight pattern change occurred in EGS, with more than 65% of the study region experiencing an advancing trend in EGS (Fig 4*b*). A notable change occurred in LGS, which became about 80% longer (over 0.5 days yr^-1^) in northern America and Eurasia (Fig 4*c*), which was similar to BGS trend. Results indicated that a longer LGS trend is mainly caused by a significant advance in spring phenology during 1982–1992, which is consistend with previous studies [17], [41]. During 1992–2002, BGS advanced throughout most of the hemisphere, with exceptions being parts of central northern America and southern Asia, while EGS was delayed throughout almost the entire hemisphere, resulting in a longer LGS (Fig 4*e*). During 2002–2012, a marked advancing trend (over 0.8 days yr^-1^) in BGS occurred form 55°N to 75°N (Fig 4*i*), correspondingly, the LGS can also be found to be siginificantly prolonged in this region (Fig 4*m*). From 1982 to 2012, unlike BGS, EGS and LGS, PGS showed a more fragmented pattern, but overall there was not a significant trend in PGS. From the point of spatial patterns, PGS was more relvant with BGS than EGS or LGS (Fig 4*d*, 4*h* and 4*m*).

**Fig 5:**
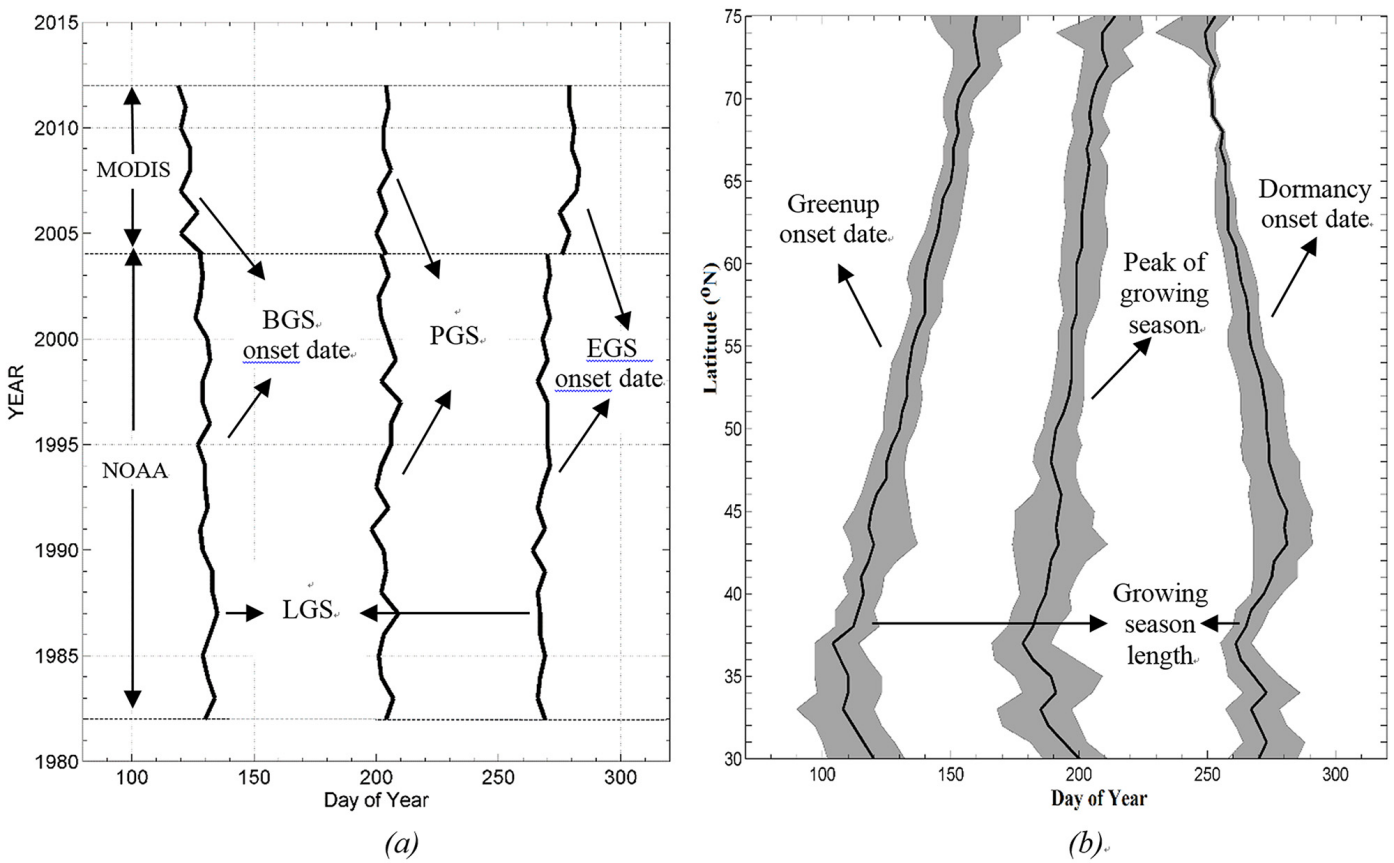
Overall trends of phenological transitional dates (DOY, day of year) along the longitudinal gradient in Northern Hemisphere during 1982–2012. (*a*) overall trends in dates of vegetation phenology for 1982–2012. (*b*) variability of vegetation phenology along the latitudinal gradient from 1982 to 2002.The dark solid line indicates the mean and the shaded zone indicates the range of interannual variability.

Fig 5 shows the overall trends of phenological metrics changes during 1982–2012 and interannual variability of vegetation phenology along the latitudinal gradient. As seen in Fig 5*a* and Table 1, changes in BGS, EGS, LGS and PGS showed high interannual variations. Long-term variations in BGS, EGS and LGS showed ‘earlier green-up onset’, ‘delayed dormancy onset’, and ‘prolonged growing season’ features, which are consistent with previous studies [2],[10],[17]. Only using NOAA NDVI data, linear regression between phenological timing and time indicates that the rate of change is -0.19 days per year (R^2^ = 0.33, P < 0.001) for BGS, 0.21 days per year (R^2^ = 0.41, P < 0.001) for EGS, 0.32 days per year (R^2^ = 0.44, P < 0.001) for LGS and 0.01 days per year (R^2^ = 0.00, P < 0.001) for PGS for the period of 1982–2002 over the Northern Hemisphere. For using MODIS NDVI data, the rate of change is -0.50 days per year (R^2^ = 0.25, P < 0.001) for BGS, 0.35 days per year (R^2^ = 0.16, P < 0.001) for EGS, 0.59 days per year (R^2^ = 0.38, P < 0.001) for LGS and 0.05 days per year (R^2^ = 0.01, P < 0.001) for PGS for the period of 2002–2012. As seen in Fig 5*b*, the vegetation phenology was strongly dependent on latitude, which indicates temperature is the primary controlling factor on vegetation phenology. However, it should be noted that phenological metrics have large variations along the longitudinal gradient (see the interannual variability in Fig 5*b*), which reflects the vegetation phenology response to global warming for different latitude. Linear regression between phenological timing and latitude indicates that the rate of change is 1.24 days per degree of latitude (R^2^ = 0.96, P < 0.001) for BGS, -0.51 days (R^2^ = 0.57, P < 0.001) for EGS, -2.02 days (R^2^ = 0.97, P < 0.001) for LGS and 0.54 days (R^2^ = 0.76, P < 0.001) for PGS in Northern Hemisphere between 30° N and 75° N.

**Table 1:** Shifts of phenology metrics based on NOAA AVHRR data (1982–2002) and MODIS data (2002–2012) (days per decade).

Phenological metric	NOAA (1982–2002)			MODIS (2002–2012)		
	Shift	*R*^*2*^	*P*	Shift	*R*^*2*^	*P*
BGS	-0.193	0.313	0.002	-0.591	0.285	0.052
EGS	0.205	0.39	0.001	0.291	0.042	0.261
LGS	0.313	0.41	0.000	0.618	0.417	0.012
PGS	0.002	0.0008	0.988	0.164	0.086	0.381

**Fig 6:**
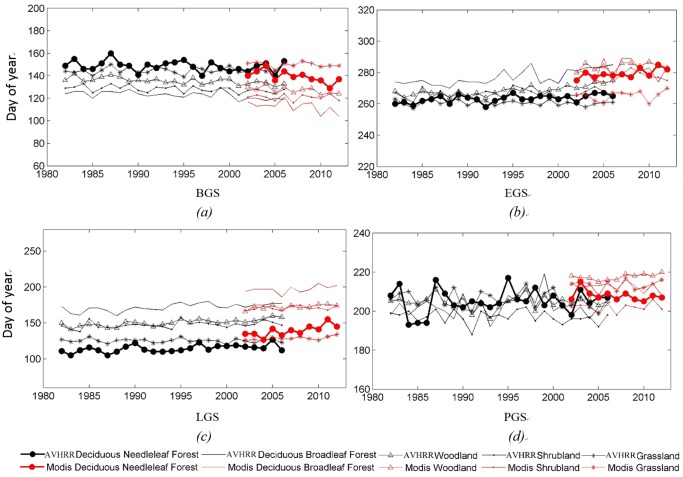
Interannual variations of (*a*) BGS, (*b*) EGS, (*c*) LGS and (*d*) PGS for specific vegetation classes over Northern Hemisphere based on AVHRR data (back lines, 1982–2006) and MODIS data (red lines, 2002–2012).

The variation in mean BGS, EGS and PGS for specific vegetation classes were estimated by using AVHRR data and MODIS data. Fig 6 provides a temporal depiction of the variation in BGS, EGS, LGS and PGS for specific vegetation classes during 1982–2012. It was found that deciduous broadleaf forest and woodland exhibited a higher rate of change for both BGS (−0.24 and −0.25 days yr^−1^, respectively) and EGS (+0.48 and +0.33 days yr^−1^, respectively) than the other classes. Shrubland showed the least variation in BGS (-0.08 days yr^−1)^ and EGS (0.05 days yr^−1^) compared to the other classes. Interestingly, including deciduous broadleaf forest, woodland and deciduous needleleaf forest, the delay in EGS is consistently larger than the advancement in BGS,which leads to the inference that autumnal response of vegetation is greater and faster than that in spring, which is consistent with the findings of previous studies [42]. All these specific vegetation classes revealed a prolonging growth cycle with earlier BGS and delayed EGS.

### Variation in Phenology within Different Holdridge Life Eco-Zones

In order to examine the phenological trends and their characteristic differences, the index *RPS* was calculated for the Holdridge life eco-zones in the study area. The index *RPS* was divided arbitrarily into four groups based on the value of the change rate of phenology shift. The first group represented the slight mode of phenological shift, in which the value of *RPS* was from 0.2 to 0.5 or from -0.5 to -0.2. The second group represented the moderate mode where the *RPS* was from 0.5 to 0.8 or from -0.8 to -0.5. The third group belonged to the fast mode with the *RPS* from 0.8 to 1.0 or from -1.0 to -0.8, and the group four was for the acute mode with the vulue of *RPS* above 1.0 or below -1.0.

**Fig 7:**
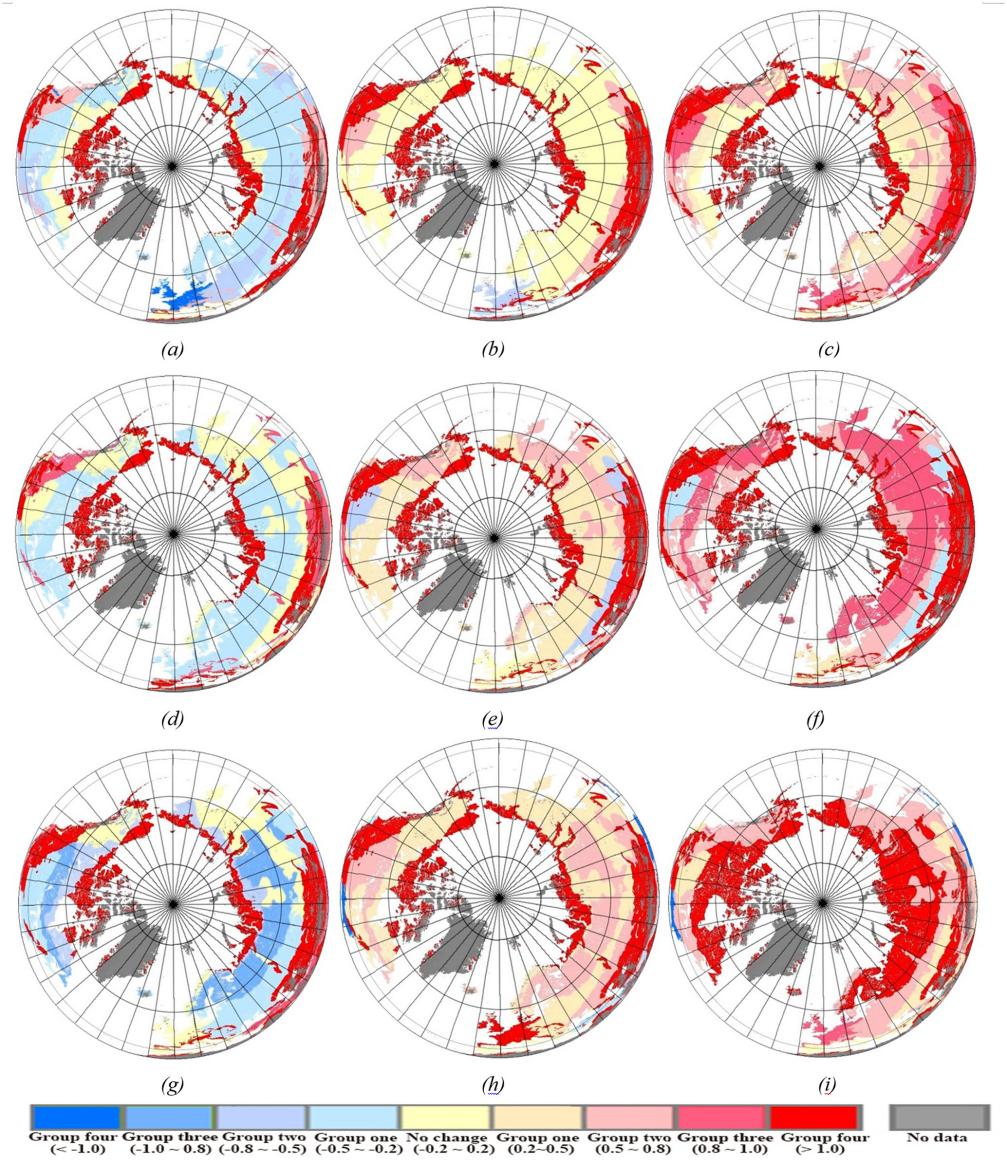
Spatial distribution of the change rate of phenology shift (*RPS*) within different Holdridge life eco-zones during the growing season from 1982 to 2012 in the Northern Hemisphere. Positive values (red colors) represent later onset (BGS), later finish (EGS), longer duration (LGS) of phenology in a time series. (*a*), (*d*), (*g*): *RPS* of BGS for 1982–1992, 1992–2002 and 2002–2012, respectively. (*b*), (*e*), (*h*): *RPS* of EGS for 1982–1992, 1992–2002 and 2002–2012, respectively. (*c*), (*f*), (*i*): *RPS* of LGS for 1982–1992, 1992–2002 and 2002–2012, respectively.

Fig 7 indicated that during 1982–1992, for BGS, most of the eco-zones, including boreal coniferous forest (*Ba*), boreal mountain system (*BM*), boreal tundra woodland (*Bb*), temperate continental forest (*TeDc*) and temperate steppe (*TeBSK*), were in the first *RPS* group (Fig 7*a*, a light blue area shows a slight advance trend in spring phenology). For EGS, no significant change occurred in many eco-zones (Fig 7*b*, yellow areas). Due to most of the eco-zones experienced an advancing trend in BGS, resulting in a longer growing season length. So for LGS, boreal coniferous forest belonged to the group one, boreal mountain system and temperate steppe belonged to the group two (Fig 7*c*, light red areas), all eco-zones experienced an extend trend in LGS. During 1992–2002, the same spatial pattern of *RPS* as the period of 1982–1992, but we should note that a remarkable extend occurred in the vegetation growing length, due to the ‘earlier green-up onset’, ‘delayed dormancy onset’ features in eco-zones. During 2002–2012, compared with the period of 1982–1992 and 1992–2002, the spatial pattern of *RPS* showed a more significant trend in advancing of BGS, delaying of EGS and prolonging of LGS during 2002–2012, overall shows a trend of accelerating change. Such as boreal coniferous forest eco-zone, belonged to the group three, group two and group four for BGS, EGS and LGS, respectively (Fig 7*g*, 7*h* and 7*i*). It should be noted that in some eco-zones, such as polar, temperate desert and tropical desert, due to poor vegetation cover and snow (ice) and soil contamination, the spatial pattern of *RPS* shows a meaningless trend for the whole period of 1982–2012.

### Analysis of the Relationship “Phenology-Climate” within Different Eco-Zones

**Fig 8:**
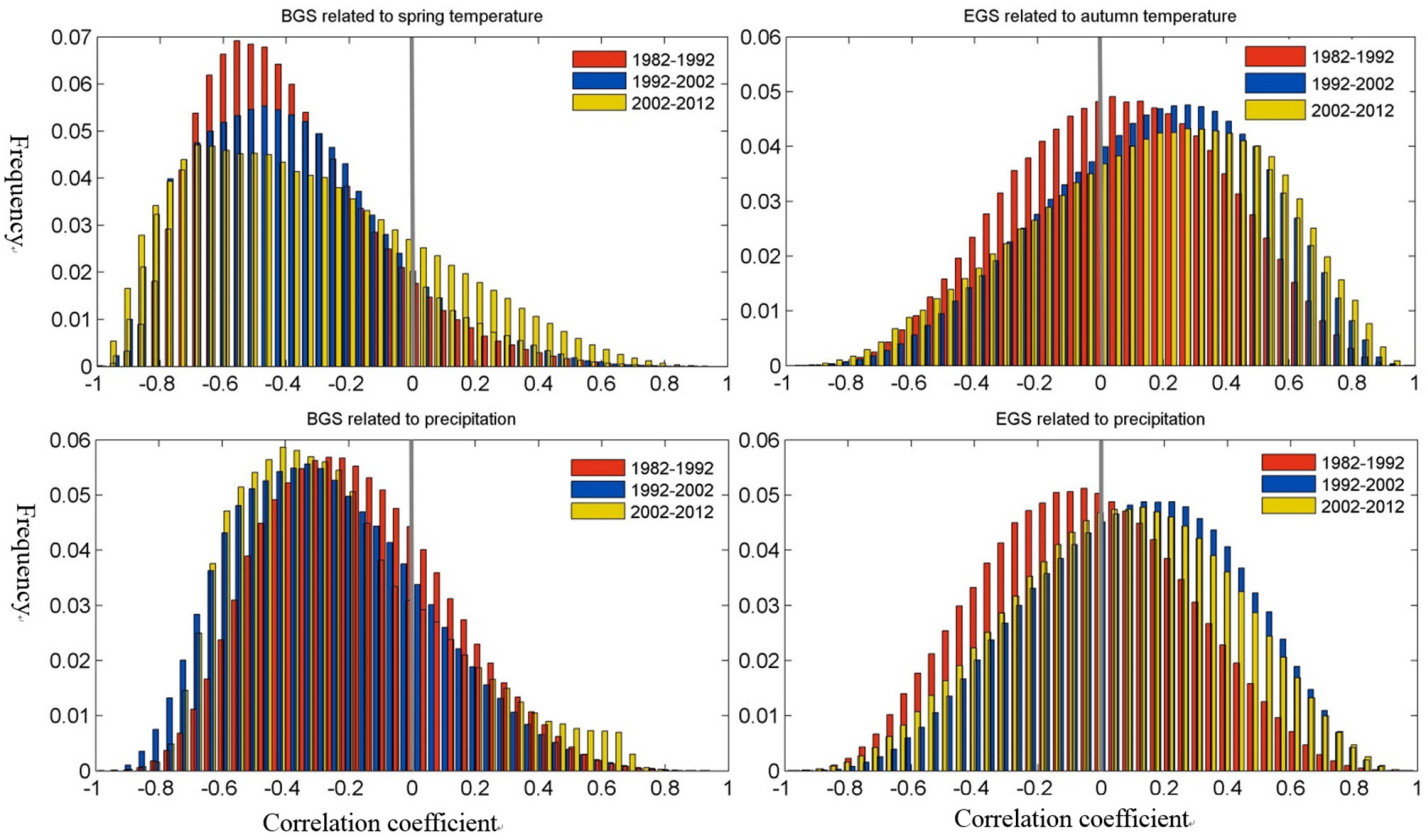
Statistical distribution of Correlation coefficients of the interannual variations of BGS, EGS and of the climatic data (spring temperature, autumn temperature and mean annual precipitation) in the Northern Hemisphere during 1982–2012.

In order to investigate the relationship between vegetation phenology and climate factors, correlation coefficients were calculated over Holdridge life eco-zones from 1982 to 2012 based on NOAA-derived phenology, MODIS-derived phenology and NCEP/NCAR reanalysis meteorological data. As we previously mentioned, in the Northern Hemisphere, phenology is primarily determined by temperature in the months period preceding the event (e.g., bud-burst, flowering, and leaf out), and higher pre-season temperatures may advance the BGS [2], [11], [17], [43], [44]. In this study, we determining the correlation analyses between the BGS and spring temperature (March, April and May), EGS and autumn temperature (August, September and October), BGS and precipitation, EGS and precipitation in the main ecozones of phenological changes. Fig 8 presents the statistical distributions of the correlation coefficients derived from Pearson correlation analyses between the interannual variations of BGS, EGS and of the climatic data (spring temperature, autumn temperature and mean annual precipitation). The presentation of the pixels were restricted to those eco-zones (*Ba*, *Bb*, *BM*, *TeBSK*, *TeDc*) only where the *RPS* exhibited a significant trend in the period 1982–2012. About 75% of all pixels showed a negative correlation with significance at p<0.05 level between the interannual variations of BGS and of the spring temperature, with Pearson’s correlation coefficient ranging from -0.97 to -0.20, and histogram peak ranging from 0.4 to 0.6, which indicates that there is a strong negative correlation between BGS and spring temperature. About 45% of all pixels exhibited a positive correlation with significance at p<0.05 level between EGS and autumn temperature, with Pearson’s correlation coefficient ranging from 0.2 to 0.95, and histogram peak ranging from 0.1 to 0.4, which indicates that there is a strong positive correlation between EGS and autumn temperature.This is consistent with previous studies that the earlier BGS and delayer EGS are the function of mean or accumulated temperatures over the Northern Hemisphere [2], [11], [45], [46]. Like the distribution pattern of the correlation coefficients between BGS, EGS and temperature, about 65% of all pixels showed a negative correlation between BGS and mean annual precipitation, with Pearson’s correlation coefficient ranging from 0.2 to 0.90, and histogram peak ranging from 0.2 to 0.4. However, overall EGS and mean annual precipitation did not exhibit a significant correlation. It should be noted that comparing the correlation coefficient of vegetation phenology and temperature with that of phenology and precipitation, we can see the correlation between temperature and phenology is much higher than that between precipitation and phenology, displaying that temperature is a major determinant of changes in vegetation phenology. This is consistent with previous studies reporting the cycles of vegetation in temperate zones are primarily controlled by temperature [2], [17], [44], [47–48].

**Fig 9:**
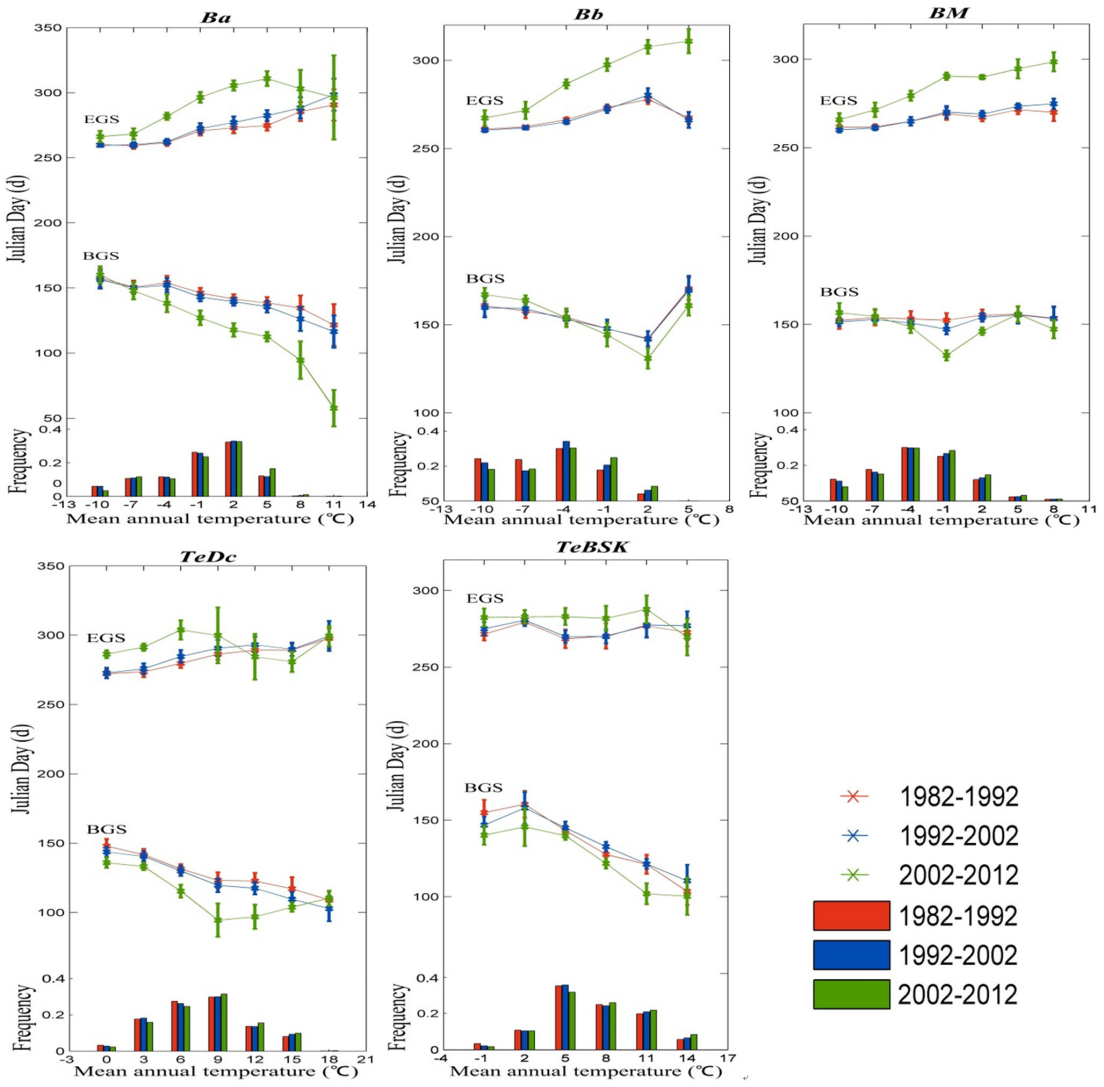
Distribution of BGS, EGS changes in relation to the mean annual temperature (upper), and the frequency of pixel in relation to mean annual temperature (bottom) within the different Holdridge life eco-zones during 1982–2012. We used the 30-year mean annual temperature to represent the climatic temperature condition. Only pixels that BGS and EGS related to temperature for statistically significant at 90% confidence level are included. (Holdridge life eco-zones including boreal coniferous forest (*Ba*), boreal tundra woodland (*Bb*), boreal mountain system (*BM*), temperate continental forest (*TeDc*), and temperate steppe (*TeBSK*)).

Even though temperature is considered as the major determinant for vegetation phenological change, little is known on vegetation phenological responses to temperature changes [44], [49–50]. Moreover, what is the phenological change per unit temperature. To further examine the temperature-sensitivity of the BGS and EGS, we analyzed the statistical distribution pattern of BGS and EGS changes in relation to mean annnual temperature within the different Holdridge life eco-zones (Fig 9). In those eco-zones whose *RPS* showed a significant trend during 1982–2012, the vegetation phenology was strongly dependent on temperature, in general, which indicates a marked advancing trend in BGS and a delaying trend in EGS along increasing temperature gradient. Interestingly, in all eco-zones, the BGS and EGS exhibited the largest variation in the temperature range of 5–12°C, which shows the vegetation phenological response to global warming is more sensitive in this temperature zone. Furthermore, in Ba, TeDC and TeBSK eco-zones, the advancement in BGS was larger than the delay in EGS, which leads to the inference that vernal vegetation response to temperature is greater and faster than that in autumn in these eco-zones. In the BM eco-zone, by contrast, the delay in EGS is consistently larger than the advancement in BGS, which also leads to the inference that autumnal vegetation response to temperature is greater and faster than that in spring in this zone. These inferences require detailed ground verification.

**Fig 10:**
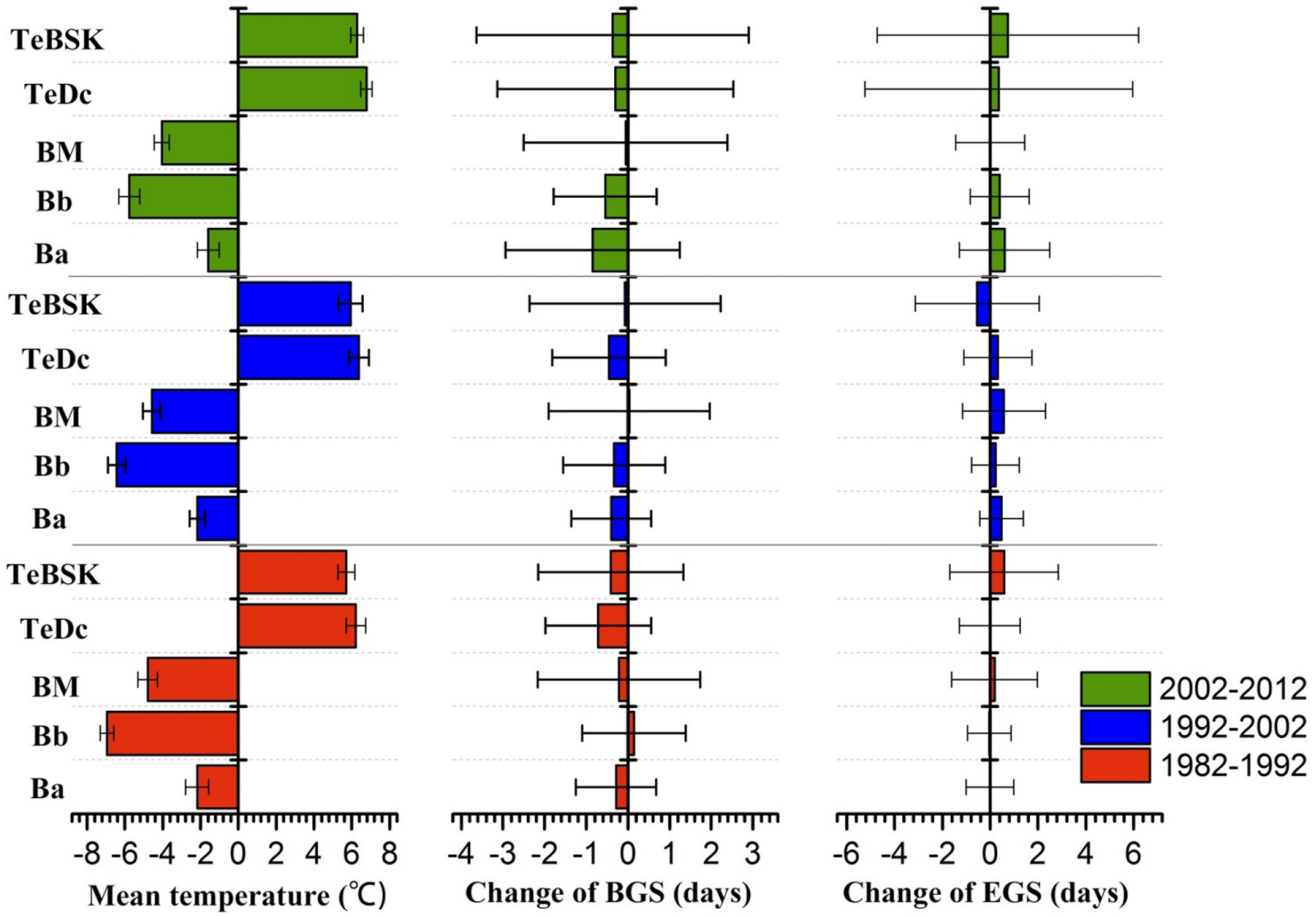
Sensitivity of vegetation phenological changes to temperature change within the different Holdridge life eco-zones during 1982–2012. The 30-year mean annual temperature was used to represent the climatic temperature condition. Only pixels that BGS and EGS related to temperature for statistically significant at 90% confidence level are included.

We next examined the vegetation phenological changes associated with temperature change within the different Holdridge life eco-zones (Fig 10). We found in all cases, higher mean temperature change associated with earlier mean BGS, such as during 2002–2012, the two largest advancement in BGS were found within the Ba (−0.84 days yr^−1^) and Bb (−0.55 days yr^−1^) zones, whose standard deviations of temperature change were 0.58 and 0.56, respectively. During 1992–2002, the largest advancement in BGS was found within the TeDc zone (−0.46 days yr^−1^), whose standard deviation of temperature change was 0.53.During 1982–1992, the largest advancement in BGS was found within the TeDc zone (−0.71 days yr^−1^), whose standard deviation of temperature change was 0.52. This is consistent with the results of previous studies showing that higher temperature-sensitivity associated with earlier onset of spring phenological events [44], [51–52]. Furthermore, in all eco-zones, overall vegetation phenology revealed a longer phenological cycle with earlier BGS and delayed EGS, which represents an extension of the growth cycle. Specifically, when comparing BGS with EGS, although the advanced BGS and delayed EGS have been reported to be the key factors that cause prolonged vegetation growing seasons, but in our study, the earlier BGS was found to be a more important factor that causes the LGS changes during 1982–2012, which differs from the results of *Jeong et al*.[2].

## Discussion

### Uncertainties in Remote Sensing Estimates on Phenological Metrics

Satellite-based techniques have been widely used for phenological monitoring and eco-environmental evaluation. However, there were many uncertainties in the results due to the temporal, spatial, and ecological complexity of vegetation biochemical processes. In this study, the uncertainties in the phenological results are twofold: uncertainties related to the different datasets and uncertainties related to the methods for determing phenological metrics.

1. *Uncertainties related to the different datasets*. In this paper, the uncertainties related to datasets include five ways: satellite drift, sensor differences, calibration uncertainties, atmopsheric effects and sub-pixel clouds [10], [25–27]. For example, sensor differences, such as NOAA AVHRR sensor or MODIS sensor, with different sensor characteristics and spatial resolution (e.g., AVHRR and MODIS have different bandwidths and central wavelengths, different number of bands and radiometric resolution; and MODIS sensor has more better onboard geometric and radiometric calibrations than AVHRR sensor), can affect the quantification of phenology shifts. Some previous studies also show that the phenology paremeters (e.g., BGS, EGS) from the different datasets were significantly different over the nothern high lattitudes [20], [23–24], [53]. Furthermore, some studies have found that the newer generation sensors such as MODIS and SPOT performed better than AVHRR for investigating the phenology in alpine vegetation [20], [24], [54]. We found similar results with AVHRR and MODIS sensor, for instance, the BGS and EGS values appear big differences in both satellite sensors (see Figs 6 and 7), but the trends of BGS and EGS show generally similar spatio-temporal patterns and good agreement between the two sensors. To reduce the uncertainty of the results, in this paper the whole study period was divided into three periods (1982–1992, 1992–2002 and 2002–2012, respectively), in each period we only use the same dataset for trend analysis, thus these disagreements between AVHRR and MODIS sensors did not influence the results reported in this paper.
2. *Uncertainties related to the methods for determing phenological metrics*. On a hemispherical scale, satellite-based studies have been demonstrated to be useful for phenological monitoring. However, there are many uncertainties in the results when determing phenological metrics due to the temporal, spatial, and ecological complexity of phenological processes. Over the past few years, several models have been developed for detecting the phenological metrics by using satellite data, including NDVI thresholds, largest NDVI increase, backward-looking moving averages, and fitting logistic functions, but each model has its uncertainty for estimating the phenological metrics. Overall, comparative studies have shown that the Midpoint_pixel_ threshold model based on variations of an NDVI_ratio_ is the one of the most consistent methods for determing phenological metrics over a variety of ecosystems and corroborates with field-based observational data [7], [55]. Thus, in order to determine the global phenological metrics based on AVHRR and MODIS data, we used the Midpoint_pixel_ threshold method in this study. Furthermore, in response to concerns over the validity of satellite derived phenology, our research adopted a strict approach to the selection of pixels for analysis through statistical tests of significance at different levels.

### Influencing Factors of Phenological Shifts

Many factors affect phenological shift patterns, such as topographic conditions, meteorological conditions, human disturbance and structural geology, among other factors. As far the global vegetation phenology, as reported by the IPCC’s AR4 [56], many natural environmental factors strongly influence phenological shifts, such as temperature, precipitation/soil moisture, photoperiod, winter chilling. As we previously mentioned, in the Northern Hemisphere, phenology is primarily determined by temperature, specifically, alpine plant species are considered to be the most sensitive vegetation ecosystem in response to rising temperature. It should be noted that different plant species have different temperature requiements for development, also different plant species have different responses to temperature change. For instance, *Jin et al*. [57] reported that on the Tibetan Plateau, 1°C increase in soil temperature will advance the BGS by 4.6 to 9.9 days, and postpone the EGS by 7.3 to 10.5 days among the different plant species. Meanwhile, our study also indicates that there were different plant phenological responses to temperature within different eco-zones. In particular, our study further revealed that higher temperature warming related to earlier onset of BGS.

Even though temperature clearly played the most important role in vegetation phenology at the high latitudes, precipitation/soil moisture was also an important factor for vegetation phenology. Especially in arid and semi-arid regions, precipitation/moisture was a more important factor in vegetation phenology. For example, *Prieto et al*. [58] found that flowering time was strongly influenced by precipitation patterns in Mediterranean shurbs. *Lesica et al*. [59], *Broich et al*. [60] and *Krishnaswamy et al*. [15] also reported that precipitation and moisture may play a more important role in phenological cycles in arid and semi-arid systems. In the high-latitude regions snowmelt is also an important factor affecting alpine vegetation phenology, especially in some cold regions, the time of snowmelt is often considered to be the observable indicators of phenological events in tundra plants [61]. Some recent studies have found that the spatial pattern of vegetation growing season mainly corresponded to the snowmelt gradient and plant species in the alpine ecosystem [62–63]. *Jeganathan et al*.[42] also found some link between the trend in the onset of snowmelt and inter-annual variation in BGS and EGS. Interestingly, in some snow-rich regions, such as the Kola Peninsula in Russia, and outer Troms County in northern Norway, mountain birch and herbaceous plants may have green leaves before snowmelt in spring when there is enough light through the snow [64–65]. Overall, in some high-latitude alpine areas, timing of snowmelt and temperature are the two most important factors controlling phenology.

In addition to temperature and precipitation, the photoperiod and winter chilling also have an influence on vegetation life cycle at the high latitudes. Although it is commonly assumed that winter chilling plays a important role in regulation of phenological phases, in fact, the interactions between chilling and photoperiod might play a more important role in controlling phenological phases at the high latitudes. For example, some recent experiments suggest that phtotoperiod might play an important role in controlling budburst and scenescence [66]. Some previous studies found that warmer temperatures during the winter will slow the dormancy breaking process by delaying the fulfillment of the chilling requirement. However, warm temperatures during the spring will advance the plant life growth by accelerating the accumulation of heat. [45], [52], [67]. Especially for humid extratropical areas, phenology is more controlled by winter chilling, photoperiod and temperature [68–69].

## Conclusions

During the period from 1982 to 2012, most of the high-latitude regions experienced significant trends in advancing of BGS, delaying of EGS and prolonging of LGS, but no significant trends in PGS. Comparing the period of 2002–2012 with the periods of 1982–1992 and 1992–2002, the spatial pattern of RPS shows a more significant trend in advancing of BGS, delaying of EGS and prolonging of LGS during 2002–2012, overall shows a trend of accelerating change. The correlation coefficients between climate and phenological events for the Holdridge life eco-zones from 1982 to 2012 showed that temperature was a major determinant of changes in vegetation phenology, with a significant advanced trend in BGS and a delayed trend in EGS along an increased temperature gradient. However, the phenological response of vegetation to temperature varied across different eco-zones. This study has shown that Midpointpixel threshold method is useful, and the trends in this study are generally in agreement with trends in previous studies. However, there are considerable uncertainties associated with the data and methods. To reduce uncertainies, it is clearly needed to design a biogeochemical model that integrates satallite-derived parameters, plant types, natural environmental factors and ground-based observations in future research.
